# Women's Holidays

**Published:** 1919-07-26

**Authors:** Francis M. Wyndham, Helen A. Pownall, A. F. London, J. Scott Lidgett


*To the Editor of* The Hospital.

### Women's Holidays.

*To the Editor of* The Hospital.

Sir,—Never before, perhaps, in the history of the Women's Holiday Fund have holidays been so much needed and desired. Already applications have been received from women who have not had a holiday for twenty or thirty years, in some cases from those who have never had one in their lives. Others plead to be allowed a week at the sea to enable them to get through their daily round of hard work and drudgery during the rest of the year. It is for these women—those who cannot afford a holiday without the help given by this Fund—that we make our appeal. In 1913 1,240 women and 155 babies were sent away, but during the last five years, owing to the high prices and the heavy railway fares (with the 50 per cent. increase), the work of the Society has been sadly limited. A speedy and generous response to this appeal from those who are now enjoying or planning their own holidays will, however, enable the committee to send away a larger number of women than seems at present possible. Donations will be gratefully acknowledged by Miss Cooper, secretary, Women's Holiday Fund, 76 Denison House, Vauxhall Bridge Road, S.W. 1; the treasurer, A. S. Daniell, Esq., Fairchildes, Warlingham, Surrey; or by Mrs. Frank Pownall, 8 Ashley Place, S.W. 1.

A. F. London, Francis M. Wyndham,

J. Scott Lidgett, Helen A. Pownall.

